# A Semi-Supervised Domain Adaptation Method for Sim2Real Object Detection in Autonomous Mining Trucks

**DOI:** 10.3390/s25051425

**Published:** 2025-02-26

**Authors:** Lunfeng Guo, Yinan Guo, Jiayin Liu, Yizhe Zhang, Zhe Song, Xuedong Zhang, Huajie Liu

**Affiliations:** 1 School of Mechanical and Electrical Engineering, China University of Mining and Technology (Beijing), Beijing 100000, China; bqt2000401001@student.cumtb.edu.cn (L.G.); jyliu@student.cumtb.edu.cn (J.L.); bqt2000401002@student.cumtb.edu.cn (Y.Z.); sqt2200401026@student.cumtb.edu.cn (Z.S.); sqt2200401034@student.cumtb.edu.cn (X.Z.); 2 Mining University (Beijing) Inner Mongolia Research Institute, China University of Mining and Technology (Beijing), Ordos 017000, China; 3 Key Laboratory of Smart Mining and Robotics, Ministry of Emergency Management, Beijing 100000, China; 4 China Academy of Safety Science and Technology, Beijing 100000, China; 5 Suzhou Automotive Research Institute (Wujiang), Tsinghua University, Suzhou 215200, China; liuhuajie@tsari.tsinghua.edu.cn

**Keywords:** autonomous driving truck, semi-supervised domain adaption, active domain adaption, open-pit mine, object detection

## Abstract

In open-pit mining, autonomous trucks are essential for enhancing both safety and productivity. Object detection technology is critical to their smooth and secure operation, but training these models requires large amounts of high-quality annotated data representing various conditions. It is expensive and time-consuming to collect these data during open-pit mining due to the harsh environmental conditions. Simulation engines have emerged as an effective alternative, generating diverse labeled data to augment real-world datasets. However, discrepancies between simulated and real-world environments, often referred to as the Sim2Real domain shift, reduce model performance. This study addresses these challenges by presenting a novel semi-supervised domain adaptation for object detection (SSDA-OD) framework named Adamix, which is designed to reduce domain shift, enhance object detection, and minimize labeling costs. Adamix builds on a mean teacher architecture and introduces two key modules: progressive intermediate domain construction (PIDC) and warm-start adaptive pseudo-label (WSAPL). PIDC builds intermediate domains using a mixup strategy to reduce source domain bias and prevent overfitting, while WSAPL provides adaptive thresholds for pseudo-labeling, mitigating false and missed detections during training. When evaluated in a Sim2Real scenario, Adamix shows superior domain adaptation performance, achieving a higher mean average precision (mAP) compared with state-of-the-art methods, with 50% less labeled data required, achieved through active learning. The results demonstrate that Adamix significantly reduces dependence on costly real-world data collection, offering a more efficient solution for object detection in challenging open-pit mining environments.

## 1. Introduction

Object detection technology is the key to enabling safe and efficient path planning and navigation for autonomous trucks in open-pit mining [1,2]. Unlike urban driving, the open-pit mining environment is complex, with unstructured road boundaries, variable conditions, and challenges such as mud, gravel, and dust. Additionally, frequent bad weather events like sandstorms, dense fog, and snow or rain further increase the demand for the generalization ability of object detection algorithms [2,3]. Therefore, training data must cover diverse conditions, including various weather and lighting scenarios and equipment types, to ensure stable detection performance. However, collecting data under extreme conditions is extremely difficult due to the scarcity of data in poor weather and working conditions and the high risk associated with operating in complex environments, increasing the difficulty and cost of data collection. Moreover, accurately labeling data in these unstructured complex scenarios is time-consuming and labor-intensive, further delaying algorithm training and deployment [2,4].

To meet the challenges of large-scale real data acquisition and labeling, simulation engines have become an important alternative for training object detection algorithms [5,6]. These engines can generate diverse and customizable virtual scenarios and automatically produce high-quality labeled data [7,8], especially for extreme weather or hazardous conditions. For instance, a simulation platform for open-pit mines can simulate various weather conditions, road environments, and mining equipment to build comprehensive datasets for object detection models [6]. However, simulation environments often simplify the complexity of the real world, omitting details like light variations, the reflective properties of materials, and environmental disturbances, resulting in a domain shift between simulated and real data. When models are trained using simulated data, this shift hinders performance in real scenarios, making it challenging to meet robustness and safety requirements for autonomous trucks in open-pit mining [9]. To address this issue, this study focuses on improving the adaptability of simulation data to real scenarios and solving the key challenge of domain shift, significantly reducing the cost of data annotation, reducing real data dependence, and facilitating the efficient deployment and practical application of autonomous trucks in open-pit mining areas.

**Figure 1:**
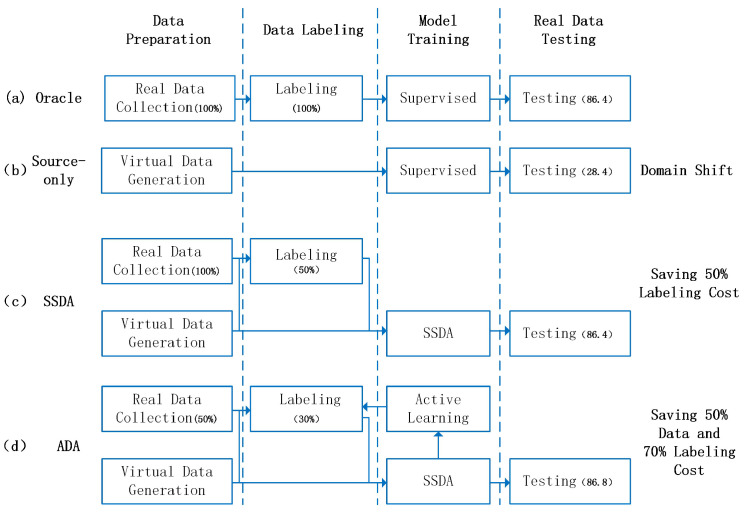
Process and performance comparison of different training strategies for detection algorithms used in autonomous driving trucks. (**a**) Oracle: this is the most commonly used algorithm training method, which relies solely on real data collection and labeling for supervised learning, achieving a test mAP of 86.4. (**b**) Source-Only: this approach depends entirely on virtual data generation without using real data and is a widely used baseline method in domain adaptation tasks, resulting in a test mAP of 28.4 due to domain bias. (**c**) Semi-supervised domain adaptation (SSDA): this is the method proposed in this study. It combines 100% real data collection with 50% labeling alongside virtual data generation, reducing labeling costs and achieving a test mAP of 86.4. (**d**) Active domain adaptive (ADA): this extended method, proposed in this study, reduces both data collection and labeling costs by 50% and 70%, respectively, using 50% real data collection, 30% labeling, virtual data generation, and active learning, achieving a test mAP of 86.8.

Domain adaptation for object detection (DA-OD) [10,11,12] methods are crucial to reducing domain shift. These methods enhance the performance of object detection algorithms in new domain environments by improving the model’s ability to generalize across different data distributions. DA-OD includes approaches such as unsupervised domain adaptation (UDA-OD), active domain adaptation (ADA-OD), and semi-supervised domain adaptation (SSDA-OD), as depicted in Figure 1. While UDA-OD [13,14] has been theoretically explored, its practical application is often limited due to the lack of labeled information in the target domain. ADA-OD [9,15,16], on the other hand, focuses on selecting and labeling the most valuable target domain samples while utilizing data from the source domain, partially labeled target domain, and unlabeled target domain. However, current ADA-OD methods mainly emphasize sample selection, with limited research on how to effectively leverage all three data types, which is the core focus of SSDA-OD. Therefore, we focus on applying SSDA-OD to better utilize all three types of training data, thus advancing the capabilities of current ADA-OD methods for object detection in new domain environments.

SSDA methods for classification [17,18] and semantic segmentation [19,20,21,22] effectively mitigate the high labeling costs and limited data diversity that challenge fully supervised training; thus, they have been extensively studied and successfully applied in fields such as autonomous driving [19,20], remote sensing [23] and medical imaging [21,22]. In particular, refs. [19,20] investigated SSDA for semantic segmentation in autonomous driving scenarios under simulated-to-real and varying weather conditions, demonstrating that minimal target domain annotations can outperform fully supervised approaches, highlighting significant practical value. Despite these promising advances, studies on semi-supervised domain adaptation for object detection remain relatively limited, highlighting a significant opportunity for further investigation.

Existing SSDA methods include image translation [24,25], domain adversarial learning [12], self-training [26], consistency regularization, and mean teacher [10,13,14] approaches. Among these, the mean teacher method is widely adopted due to its integration of consistency regularization, self-training, and teacher–student models. However, the use of this method often leads to a bias toward the source domain, primarily because the labeled source data significantly outnumber the target data, and frequent training on the limited labeled target data can result in overfitting. Additionally, mean teacher models often use fixed thresholds for pseudo-label selection [10,13], which biases the model toward easy-to-classify categories and reduces performance on challenging categories.

Previous studies introduced phased training strategies [27,28], multiple teacher models, and mixup and adaptive threshold techniques [29,30] to address these issues. However, these methods still have certain limitations. Phased training, for example, attempts to separate the use of source and labeled target data, but this limits the benefits of using different types of data simultaneously. Furthermore, some approaches, like using multiple teacher models [21,31] or co-training strategies [32], increase model complexity. Additionally, some methods apply mixup to data from different domains to mitigate source domain bias, but they typically rely on a random mixup ratio that does not adapt to evolving data requirements at different stages of training. Moreover, although adaptive threshold strategies dynamically adjust selection thresholds, starting with a preset threshold may lead to false or missed detections in early training. Consequently, developing more robust methods for effectively leveraging source data, labeled target data, and unlabeled target data while also mitigating threshold-setting issues remains a key focus for advancing SSDA-OD performance.

To this end, we propose a progressive intermediate domain construction (PIDC) strategy to improve data diversity, prevent the overfitting of labeled target and source bias, and facilitate the transition to the unlabeled target domain. This involves gradually increasing the mixup ratio when performing unlabeled target data mixup [33] with source and labeled target data. Additionally, we propose a warm-start adaptive pseudo-label (WSAPL) strategy to provide reliable initial values for adaptive threshold adjustment, reducing missed and false detection issues in early training and enhancing overall domain adaptation performance.

The three main contributions of this paper are as follows:

1. A progressive mixup strategy is introduced to effectively mitigate model bias in the source domain and overfitting on labeled target data by constructing intermediate domains.
2. A class-wise adaptive pseudo-labeling threshold strategy is proposed, in which the threshold is initialized through a warm-start mechanism. This approach reduces missed and false detections in the early training stages, significantly enhancing domain adaptation performance.
3. Experimental results in a domain adaptation scenario of object detection in an open-pit mine validate that our proposed Adamix method outperforms existing methods in terms of performance. Moreover, combining this method with existing active domain adaptation methods further improves model performance, reducing dependence on real data and lowering annotation costs.

## 2. Methods

**Figure 2:**
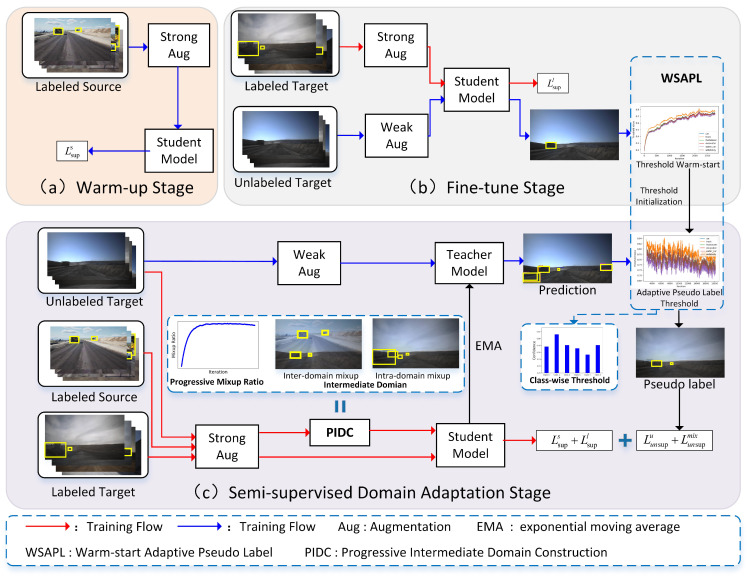
The overall architecture of the proposed Adamix framework, which consists of three training stages: (**a**) warm-up—the student model is trained on labeled source domain data to initialize feature learning; (**b**) fine-tuning—the student model is fine-tuned using labeled target domain data, while weakly augmented unlabeled data are used to initialize pseudo-labeling thresholds in the WSAPL module; (**c**) semi-supervised domain adaptation—the teacher model generates pseudo-labels for weakly augmented unlabeled data, and the WSAPL module adaptively updates these thresholds during training. In this stage, source data, labeled target data, and unlabeled target data are all used, with the PIDC module progressively constructing intermediate domains to improve training and mitigate domain bias.

As depicted in Figure 2, the Adamix framework builds on a mean teacher architecture and consists of three main stages: warm-up, fine-tuning, and semi-supervised domain adaptation (SSDA), with each stage playing a distinct role in progressively improving the model’s performance and generalization ability. The framework features two key modules: progressive intermediate domain construction (PIDC) and warm-start adaptive pseudo-label (WSAPL). PIDC gradually constructs intermediate domains by mixing source and target domain data, effectively reducing domain bias and preventing overfitting, while WSAPL initializes adaptive pseudo-label thresholds using a warm-start strategy and iteratively updates these thresholds during training to improve the reliability of the pseudo-labels.

### 2.1. Problem Definition

In the SSDA-OD task for autonomous driving trucks in open-pit mines, we use labeled simulation data as the source domain $D_{s}={\left\{\left(x_{i}^{s},y_{i}^{s}\right)\right\}}_{i=1}^{n_{s}}$ and real-world data as the target domain. The target domain consists of a small amount of labeled target data $D_{l}={\left\{\left(x_{j}^{l},y_{j}^{l}\right)\right\}}_{j=1}^{n_{l}}$ and a large amount of unlabeled target data $D_{u}={\left\{x_{j}^{u}\right\}}_{j=1}^{n_{u}}$. Here, $x_{i}^{s}$ and $y_{i}^{s}$ denote the *i*-th image and its bounding box label in the source domain, $x_{j}^{l}$ and $y_{j}^{l}$ denote the *j*-th labeled image and its label in the target domain, and $x_{j}^{u}$ represents the *j*-th unlabeled image in the target domain. $n_{s}$ and $n_{l}$ indicate the number of labeled source and the labeled target images, respectively, while $n_{u}$ represents the number of unlabeled target images. The goal is to effectively utilize the data from $D_{s}$, $D_{l}$, and $D_{u}$ to train the object detection model, enabling it to achieve better performance on test data sampled from the target domain distribution.

### 2.2. Progressive Intermediate Domain Construction

For SSDA-OD tasks, the labels of the source domain and labeled target data aid the model in learning foundational features and stabilizing early training. However, the abundance of source data can introduce bias, while the limited labeled target data may lead to overfitting. Unlabeled target data, which are often more plentiful, contain critical target domain features that are essential for enhancing model generalization. However, the absence of labels in these data leads to lower-quality pseudo-labels in the early stages, increasing the risk of model bias or divergence. Therefore, it is crucial to carefully balance the use of these three kinds of data throughout the training process to fully leverage their strengths.

To address these challenges, we propose constructing intermediate domains by applying mixup operations to the unlabeled target data and both the source domain and labeled target data. Additionally, we introduce a progressive mixup strategy (PMS) that dynamically adjusts the mixup ratio of the unlabeled target data throughout training, optimizing the use of all three data types.

#### 2.2.1. Intermediate Domain Construction via Mixup

We employ mixup to construct intermediate domains, helping the model adapt from the source domain to the target domain. Specifically, we perform mixup between the unlabeled target data and both the source data and the labeled target data. The batch sizes for the source, labeled target, and unlabeled target domains are set to the same value, bat. During each iteration, the *i*-th image in the batch from the unlabeled target domain is mixed with the corresponding *i*-th image from the source and labeled target domains to generate intermediate domain data.

***Inter-domain mixup*****:** We create inter-domain mixed samples by mixing the *i*-th source data $x_{i}^{s}$ with the *i*-th unlabeled target data $x_{i}^{u}$. The inter-domain mixed image and its corresponding label are generated as follows:

(1) $$\left\{\begin{array}{cc} x_{i,inter}^{mix} & =m\cdot x_{i}^{s}+\left(1-m\right)\cdot x_{i}^{u}\\ y_{i,inter}^{mix} & =y_{i}^{s}+{\hat{y}}_{i}^{u} \end{array}\right.$$

where *m* is the mixing ratio, $y_{i}^{s}$ is the source domain label, and ${\hat{y}}_{i}^{u}$ is the pseudo-label from the unlabeled target data.

***Intra-domain mixup*****:** Similarly, we perform intra-domain mixup by mixing the *i*-th labeled target data $x_{i}^{l}$ with the *i*-th unlabeled target data $x_{i}^{u}$. The intra-domain mixed image and its corresponding label are defined as

(2) $$\left\{\begin{array}{cc} x_{i,intra}^{mix} & =m\cdot x_{i}^{l}+\left(1-m\right)\cdot x_{i}^{u}\\ y_{i,intra}^{mix} & =y_{i}^{l}+{\hat{y}}_{i}^{u} \end{array}\right.$$

where $y_{i}^{l}$ is the label from the labeled target data.

***Intermediate domain construction*****:** Finally, we combine inter-domain and intra-domain mixed data to construct the full intermediate domain. The mixed data $x^{mix}$ and labels $y^{mix}$ are defined as

(3) $$\left\{\begin{array}{cc} x^{mix} & ={\left\{x_{i,inter}^{mix}\right\}}_{i=1}^{bat}\cup {\left\{x_{i,intra}^{mix}\right\}}_{i=1}^{bat}\\ y^{mix} & ={\left\{y_{i,inter}^{mix}\right\}}_{i=1}^{bat}\cup {\left\{y_{i,intra}^{mix}\right\}}_{i=1}^{bat} \end{array}\right.$$

where bat represents the batch size.

#### 2.2.2. Progressive Mixup Strategy

Previous studies mostly used a fixed or randomized approach when assigning values to mixup ratios [33,34,35]. This approach provides modest data augmentation and is difficult to adapt to the diverse data requirements of the model at different training stages. In the SSDA-OD task, the model requires more source data and labeled target data in the early training stage. This ensures training stability and helps the model learn key underlying features and patterns accurately. However, as training advances, the model’s generalization ability increases, and more unlabeled target data are needed to improve target domain performance and reduce bias to the source domain and the overfitting of labeled target data.

Therefore, a fixed or random mixup ratio may not fully utilize the potential of data augmentation. To address this, we introduce a progressive mixup strategy (PMS). In the $t_{s}$-th iteration of SSDA, the predicted probability vector for the *n*-th object, denoted as $q_{n,t_{s}}$, is generated by the teacher model based on the unlabeled target domain data, where $q_{n,t_{s}}\left[j\right]$ represents the predicted probability for the *j*-th class. We select the category with the highest probability as the preliminary label and use its value as the predicted confidence for this object. Based on these preliminary labels, we classify objects into different sets according to their prediction confidence. The confidence set for category *c* is defined as

(4) $$Q_{c,t_{s}}=\left\{q_{n,t_{s}}\left[c\right]|\mathrm{argmax}\left(q_{n,t_{s}}\right)=c\right\}$$

To prevent the average confidence of the teacher model’s predictions from being skewed by the high number of objects in some categories, we calculate the average confidence by selecting, for each category, the top-*k* highest confidence values with the same number of objects from the confidence set of that category. We then compute the all-class local average confidence for the $t_{s}$-th iteration $\bar{Q_{t_{s}}^{local}}$, using the following formula:

(5) $$\bar{Q_{t_{s}}^{local}}=\frac{1}{C}\sum_{c=1}^{C}\frac{1}{k}\sum_{i=1}^{k}Q_{c,t_{s}}\left[i\right]$$

where *C* is the total number of categories and $Q_{c,t_{s}}\left[i\right]$ denotes the *i*-th highest confidence value in category *c* sorted by confidence. This ensures a balanced contribution of categories to the average confidence calculation.

Next, we introduce a mechanism to dynamically update the mixup ratio. The initial mixup ratio is defined as $m_{0}=\frac{1}{C}$, and it is subsequently updated using an exponential moving average (EMA) operation.

(6) $$m_{t}=\lambda_{1}m_{t-1}+\left(1-\lambda_{1}\right)\bar{Q_{t_{s}}^{local}}$$

where $\lambda_{1}$ is a smoothing parameter used to control the fusion of old and new information. Through this progressive adjustment mechanism, the mixup ratio can adapt to the data requirements of different training stages. In the early stage, a higher ratio of source data to labeled target data helps stabilize the model. In the later stage, a higher proportion of unlabeled target data in the mixup process improves the model’s generalization ability and reduces the risks of source domain bias and overfitting the labeled target.

### 2.3. Warm-Start Adaptive Pseudo-Label

The quality of pseudo-labels is critical for the mean teacher-based SSDA-OD method because the student model learns from unlabeled data using pseudo-labels generated by the teacher model. However, selecting pseudo-labels with fixed confidence thresholds can lead to issues with varying learning difficulty across categories. Using adaptive thresholds can avoid missed and false detections, but it begins with a fixed value. We propose WSAPL to address these issues, which has two steps: warm-Start and adaptive pseudo-label.

#### 2.3.1. Warm-Start Strategy

The warm-start phase aims to calculate the initial pseudo-labeling threshold for each category before the SSDA phase. We employ a method similar to a previous work that used predictive confidence to compute these thresholds. Specifically, during the fine-tuning phase, we calculate the average confidence for each category, as well as the all-class average confidence, to prepare for the SSDA phase and determine the initial pseudo-labeling values.

In the $t_{f}$-th iteration of the fine-tuning phase, we compute the confidence set $Q_{c,t_{f}}$ of category *c* from the object confidence predicted by the student model, which is based on the predictions for the unlabeled target domain, using Equation (4).

The local average confidence of category *c* is the average confidence of the top *k* objects with the highest confidence in $Q_{c,t_{f}}$.

(7) $$\bar{Q_{c,t_{f}}^{local}}=\frac{1}{k}\sum_{i=1}^{k}Q_{c,t_{f}}\left[i\right]$$

Initially, the global average confidence for category *c* $\bar{Q_{c,t_{f}}^{global}}$ is set to $\frac{1}{C}$. Then, we update the global average confidence $\bar{Q_{c,t_{f}}^{global}}$ of category *c* at the $t_{f}$-th iteration using EMA:

(8) $$\bar{Q_{c,t_{f}}^{global}}=\lambda_{2}\bar{Q_{c,(t_{f}-1)}^{global}}+\left(1-\lambda_{2}\right)\bar{Q_{c,t_{f}}^{local}}$$

Similarly, the all-class global average confidence, $\bar{Q_{t_{f}}^{global}}$, is also initialized as $\frac{1}{C}$. This value is updated at the $t_{f}$-th iteration as follows:

(9) $$\bar{Q_{t_{f}}^{global}}=\lambda_{2}\bar{Q_{t_{f}-1}^{global}}+\left(1-\lambda_{2}\right)\bar{Q_{t_{f}}^{local}}$$

#### 2.3.2. Adaptive Pseudo-Label

At the beginning of the SSDA phase, to provide reliable initial values for adaptive threshold adjustment, we initialize the global average confidence values using those computed during the final iteration of the fine-tuning phase. Specifically, for each category *c*, we set the initial global average confidence $\bar{Q_{c,(t_{s}=0)}^{global}}$ of the category *c* to the value $\bar{Q_{c,(t_{f}=TF)}^{global}}$ calculated at the last iteration ($t_{f}=TF$) of the fine-tuning phase. Similarly, we set the initial all-class global average confidence $\bar{Q_{\left(t_{s}=0\right)}^{global}}$ to the value $\bar{Q_{\left(t_{f}=TF\right)}^{global}}$, which was computed during the same iteration. These initializations are formalized as follows:

(10) $$\bar{Q_{c,(t_{s}=0)}^{global}}=\bar{Q_{c,(t_{f}=TF)}^{global}},\;\bar{Q_{\left(t_{s}=0\right)}^{global}}=\bar{Q_{\left(t_{f}=TF\right)}^{global}}$$

Subsequently, the global average confidence of the category *c* is updated in each iteration:

(11) $$\bar{Q_{c,t_{s}}^{global}}=\lambda_{2}\bar{Q_{c,(t_{s}-1)}^{global}}+\left(1-\lambda_{2}\right)\bar{Q_{c,t_{s}}^{local}}$$

The all-class global average confidence is also updated:

(12) $$\bar{Q_{t_{s}}^{global}}=\lambda_{2}\bar{Q_{t_{s}-1}^{global}}+\left(1-\lambda_{2}\right)\bar{Q_{t_{s}}^{local}}$$

In object detection tasks, difficult-to-learn categories often have higher false detection rates. Setting lower thresholds for these categories may lower recall for other categories. To prevent this, we control the threshold difference between categories within a range. We use the all-class global confidence and the global average confidence of a specific category to calculate individual category thresholds.

The pseudo-labeling threshold $\mu_{t_{s}}\left[c\right]$ for each category *c* at the $t_{s}$-th iteration of the SSDA phase is calculated using the following equation:

(13) $$\mu_{t_{s}}\left[c\right]=\bar{Q_{t_{s}}^{global}}+\eta \bar{Q_{c,t_{s}}^{global}}$$

where η is used to balance the effects of all-class confidence and category-specific confidence on the pseudo-labeling threshold. This threshold considers both the overall average confidence and the average confidence of each category, improving the model’s filtering of pseudo-labels and adaptability.

Our method initializes with a suitable threshold, preventing the missed and false detections common in previous methods due to setting improper initial thresholds. It also adaptively adjusts pseudo-label selection criteria based on category learning progress, improving model domain adaptive performance.

### 2.4. Adamix Training Pipeline

In this subsection, we describe the procedure of training the proposed Adamix model, which is based on the classical mean teacher framework [13]. The training process is divided into three key stages: warm-up, fine-tuning, and semi-supervised domain adaptation, each with specific goals and corresponding loss functions.

#### 2.4.1. Initial Training: Warm-Up and Fine-Tuning Stages

In this subsection, we describe the initial training phase of the Adamix model, which combines the warm-up and fine-Tuning stages. This initial training aims to provide a strong feature foundation using both source domain data and a small set of labeled target domain data to reduce domain bias before moving into the semi-supervised domain adaptation stage.

The warm-up stage involves supervised training on the labeled source domain data, $D_{s}$. This stage helps the student model learn essential features from the source domain, forming a foundational basis for further training. The supervised loss $L_{sup}^{s}$ for this stage is calculated as follows:

(14) $$\begin{array}{c} L_{sup}^{s}=\sum_{i}(L_{cls}^{rpn}\left(x_{i}^{s},y_{i}^{s}\right)+L_{reg}^{rpn}\left(x_{i}^{s},y_{i}^{s}\right)\\ +L_{cls}^{roi}\left(x_{i}^{s},y_{i}^{s}\right)+L_{reg}^{roi}\left(x_{i}^{s},y_{i}^{s}\right)) \end{array}$$

where $L_{cls}^{rpn}$ and $L_{cls}^{roi}$ denote the classification losses for the region proposal network (RPN) and region of interest (ROI) modules, respectively. The primary task of the RPN is to generate candidate regions (proposals), while the ROI module refines these proposals [36]. The classification loss for the RPN is computed using binary cross-entropy, whereas the ROI’s classification loss is evaluated using multi-class cross-entropy. Additionally, $L_{reg}^{rpn}$ and $L_{reg}^{roi}$ correspond to the regression losses for the RPN and ROI modules, both employing L1 loss.

Following the warm-up stage, the fine-tuning stage is conducted on a small set of labeled target domain data, $D_{l}$. This stage is intended to reduce domain bias and enable the model to better adapt to the target domain’s characteristics. The supervised loss $L_{sup}^{l}$ for this stage is similar in form to $L_{sup}^{s}$ but is computed over the target domain data:

(15) $$\begin{array}{c} L_{sup}^{l}=\sum_{j}(L_{cls}^{rpn}\left(x_{j}^{l},y_{j}^{l}\right)+L_{reg}^{rpn}\left(x_{j}^{l},y_{j}^{l}\right)\\ +L_{cls}^{roi}\left(x_{j}^{l},y_{j}^{l}\right)+L_{reg}^{roi}\left(x_{j}^{l},y_{j}^{l}\right)) \end{array}$$

This combined initial training phase ensures that the model has a strong feature base from the source domain and is preliminarily adapted to the target domain. This setup facilitates effective semi-supervised learning in stages by reducing domain shift and enhancing generalization.

#### 2.4.2. Semi-Supervised Domain Adaptation Stage

The SSDA stage is the core of the Adamix training procedure, leveraging both labeled and unlabeled target domain data to improve the model’s adaptability to real-world scenarios. At the beginning of this stage, the parameters of the student model $\theta_{t}^{student}$ are copied to the parameters of the teacher model $\theta_{t}^{teacher}$ to ensure both models start in the same well-adapted state following the fine-tuning stage. This initialization helps the teacher model generate more reliable pseudo-labels in the early SSDA stage.

In this stage, the teacher model generates pseudo-labels for the unlabeled target domain data $D_{u}$, and the student model uses these labels for further training. The loss function in this stage combines both supervised and unsupervised losses:

(16) $$L_{total}=L_{sup}^{s}+L_{sup}^{l}+L_{unsup}^{u}+L_{unsup}^{mix}$$

where $L_{unsup}^{u}$ is the unsupervised loss for the unlabeled target domain data based on pseudo-labels generated by the teacher model. It consists of classification losses for both the RPN and ROI modules and is defined as follows:

(17) $$L_{unsup}^{u}=\sum_{j}\left(L_{cls}^{rpn}\left(x_{j}^{u},{\hat{y_{j}}}^{u}\right)+L_{cls}^{roi}\left(x_{j}^{u},{\hat{y_{j}}}^{u}\right)\right)$$

where ${\hat{y_{j}}}^{u}$ represents the pseudo-label generated by the teacher model. $L_{unsup}^{mix}$ is the unsupervised loss for the mixed intermediate domain data constructed by the PIDC module.

To maintain consistency between the teacher and student models, the teacher model’s parameters $\theta_{t}^{teacher}$ are updated using an exponential moving average (EMA) of the student model’s parameters $\theta_{t}^{student}$:

(18) $$\theta_{t}^{teacher}=\alpha \theta_{t-1}^{teacher}+\left(1-\alpha \right)\theta_{t}^{student}$$

where α is a smoothing parameter controlling the update rate.

By structuring the training process in these three stages, Adamix effectively balances the utilization of labeled data from both source and target domains while making full use of the unlabeled target data, thereby enhancing overall domain adaptation performance.

## 3. Experiments

### 3.1. Details of Implementation

In this study, we employed the faster R-CNN object detection model [36] with a VGG16 backbone, implementing all code using the Detectron2 framework. The training was conducted on two 4090D GPUs. The key hyperparameters were configured as follows: $\lambda_{1}=0.999$, $\lambda_{2}=0.99$, and η=0.1. Following the settings of the adaptive teacher method [13], we optimized the network using stochastic gradient descent (SGD) with a learning rate of 0.04 and a decay rate of α=0.9996. To ensure a fair comparison with the experimental results reported in [9], we maintained the same batch size across the source domain, labeled target domain, and unlabeled target domain, setting it to 4 for each.

During training, we first performed 10,000 iterations in the warm-up stage, followed by 25 epochs in the fine-tuning stage. Finally, we conducted 20,000 iterations in the SSDA phase.

We evaluated our method using the standard mean average precision (mAP) metric, with an IoU threshold of 0.5. A detection was considered a true positive if the intersection over union (IoU) between the predicted bounding box and the corresponding ground-truth box was at least 0.5.

For each category, the average precision (AP) was computed as the area under the precision–recall (P–R) curve:

(19) $$AP=\int_{0}^{1}P\left(R\right)\;dR$$

where P(R) represents the precision at recall *R*. The precision *P* and recall *R* are defined as follows:

(20) $$P=\frac{TP}{TP+FP},\;R=\frac{TP}{TP+FN}$$

where, TP, FP, and FN denote the numbers of true positives, false positives, and false negatives, respectively.

The overall mAP is then defined as

(21) $$mAP=\frac{1}{C}\sum_{c=1}^{C}AP_{c}$$

where *C* is the total number of classes, and $AP_{c}$ is the average precision for the *c*-th class. Using an IoU threshold of 0.5 ensures that only high-quality detections contribute to the evaluation, providing a reliable measure of detection performance.

### 3.2. Datasets

We used the Virtual-Mine and Actual-Mine datasets [9] to test our SSDA-OD method in open-pit mines. Virtual-Mine is a simulation dataset and Actual-Mine is a real dataset; both were constructed previously by our team. We created a virtual-to-real domain adaptation scenario using these datasets to assess the effectiveness of our method.

***Virtual-Mine dataset*****:** Generated using PMWorld [6], a simulation platform for open-pit mines, the Virtual-Mine dataset provides virtual images for autonomous driving trucks under various weather and lighting conditions. The platform also generates semantic segmentation and object bounding box information for each image. This dataset includes 5000 labeled images, featuring six types of mine-specific objects in different environments (i.e., a car, truck, pushdozer, excavator, water car, and wide body).

***Actual-Mine dataset*****:** The Actual-Mine dataset is based on the publicly available Automine dataset [37], in which different mine-related objects are labeled. A total of 3975 images were labeled, out of which 2975 images were used for training and 500 images were used for testing.

***Virtual-Mine to Actual-Mine domain adaptation scenario*****:** Focusing on the mine environment, this scenario evaluates the algorithm’s ability to transfer knowledge from a virtual open-pit mine environment to an actual mine environment, highlighting the challenges and needs of object detection domain adaptation in unstructured off-road environments.

### 3.3. Comparison Baseline

Our experiments extensively evaluated the proposed Adamix method by comparing it with various baseline methods. Their details are provided below.

***Source-Only***: This baseline uses only source data for training and is then tested on the target domain. It serves as a fundamental reference for understanding the impact of the domain shift.

***AT-UDA*** [13]: This method employs the classic mean teacher architecture within the adaptive teacher framework for unsupervised domain adaptation (UDA). Experiments are conducted according to the original parameter settings, providing a benchmark for our method under UDA settings.

***CMT-UDA*** [10]: This method is an improvement over the AT-UDA approach, incorporating a contrastive loss to achieve enhanced domain adaptation performance.

***Supervised***: The model is fully supervised and trained using partially labeled target data at various ratios (2.5%, 5%, 7.5%, 15%, 30%, or 50%). This baseline evaluates the effectiveness of supervised learning on the target domain across different labeling scenarios.

***Co-training***: This method leverages both source data and labeled target data in a joint training setup. It helps assess the benefits of collaborative learning in improving model performance.

***AT-SSDA*** [13]: An extension of AT-UDA that incorporates labeled target data for semi-supervised domain adaptation (SSDA) alongside source and unlabeled target data, providing insight into the performance enhancements gained from additional labeled data.

***CMT-SSDA*** [10]: This method extends CMT-UDA by employing a semi-supervised domain adaptation training paradigm.

***APT-SSDA*** [9]: Derived from the adaptive parallel teacher framework, this method represents state-of-the-art SSDA-OD techniques. It serves as a critical comparison point to evaluate the effectiveness of our proposed Adamix method.

***Dual-Teacher*** [31]: An SSDA-OD method based on the Dual-Teacher framework that combines cross-domain and semi-supervised tasks. This method utilizes two teacher–student models to learn consistent and unique information, respectively, and integrates the prediction results of the two models through four co-training strategies.

***Oracle***: This baseline represents fully supervised training using all available labeled target data, reflecting the upper bound performance achievable on the target domain. It serves as a reference standard against which other methods are measured.

### 3.4. Comparison and Analysis of Results

In this section, we present a comprehensive comparison of the proposed Adamix method against various baseline and state-of-the-art methods in different experimental settings. Specifically, we began by comparing the performance under different labeling ratios to highlight the effectiveness of Adamix in different scenarios with varying amounts of labeled data. We then conducted ablation studies to determine the contribution of each component of our approach. Finally, we evaluated the effects of different pseudo-labeling and mixup strategies. Our aim was to demonstrate the robustness, efficiency, and generalization capabilities of Adamix for the SSDA-OD task through a series of quantitative analyses.

#### 3.4.1. Main Comparison Results

We first compared the performance of Adamix with various baselines under different labeling ratios (2.5%, 5%, 7.5%, 15%, 30%, and 50%). Three independent experiments were conducted for each ratio, and the mean values and variances were calculated. The results in Table 1 demonstrate that Adamix consistently outperforms the baseline methods across all labeling ratios.

The Source-Only and AT-UDA methods do not utilize labeled target data during training and perform significantly worse than the fully supervised method. Our proposed method, Adamix, shows a significant performance improvement over these two methods.

The performance of the supervised method improves significantly when the labeling ratio is increased, reaching 80.0 mAP at a 50% labeling ratio. However, this method does not utilize source data, resulting in a lower performance than the SSDA-OD methods, including Adamix, at all labeling ratios. This suggests that methods relying solely on the labeled target domain still are still limited in their generalization performance.

In the comparison of SSDA methods, the co-training, AT-SSDA, CMT-SSDA, Dual-Teacher, and APT-SSDA methods gradually improve as the labeling ratio increases. However, Adamix outperforms these methods at all labeling ratios, especially at low ratios. For instance, at a 2.5% labeling ratio, Adamix reaches 57.0 mAP, which is significantly higher than APT-SSDA’s 53.6 mAP, suggesting that it more effectively combines source, labeled target, and unlabeled target data, substantially improving model generalization over the target domain.

As the upper limit of fully supervised methods, Oracle achieves 86.4 mAP, while our Adamix method reaches this at a 50% labeling ratio, indicating that Adamix can achieve comparable results with full supervision at 50% of the labeling cost, fully demonstrating that it can save 50% of the labeling cost.

#### 3.4.2. Component Ablation Study

To understand the independent contributions of PIDC and WSAPL, we performed ablation studies. Table 2 shows that each module independently improves performance, and combining the two yields the highest mAP, verifying their complementary nature.

Specific results show that the mAP of the baseline model is 68.2. After introducing WSAPL, the mAP improves to 70.2, indicating that this strategy effectively enhances model adaptability on the target domain by adaptively adjusting the pseudo-labeling threshold. Using only the PIDC strategy improved the mAP to 69.9, demonstrating that the model’s generalization is enhanced by progressively adjusting the mixup ratio. Combining both strategies achieves an mAP of 71.6, indicating that the PIDC and WSAPL strategies complement each other, enhancing the model’s adaptability and generalization during training.

#### 3.4.3. Parameter Ablation Study

We conducted hyperparameter ablation experiments on the smoothing parameter $\lambda_{1}$ used in the PIDC strategy, the parameter $\lambda_{2}$ used in the WSAPL strategy, and the parameter η. Table 3 shows the ablation results for $\lambda_{1}$. The results indicate that the best performance is achieved when $\lambda_{1}=0.999$. Specifically, $\lambda_{1}$ controls the smoothness of the mixup ratio update in the EMA process; lower values (e.g., 0.9 and 0.99) lead to overly rapid updates, while excessively high values (e.g., 0.9999) result in overly slow updates, both of which yield suboptimal outcomes.

Table 4 presents the ablation results for $\lambda_{2}$. The experiments show that optimal performance is obtained when $\lambda_{2}=0.99$. Since the adaptive pseudo-label thresholds need to be warm-started during the warm-up phase, a relatively small value for $\lambda_{2}$ is more appropriate.

Table 5 displays the ablation results for the parameter η. The results reveal that the best performance is achieved when η=0.1, suggesting that the differences in thresholds among various categories should be constrained within a small range. Moreover, using η to balance the effects of all-class confidence and category-specific confidence on the pseudo-labeling threshold leads to further improvements compared with relying solely on category-specific confidence.

### 3.5. Visualization of Mixup Ratios and Threshold Adjustment Curves

**Figure 3:**
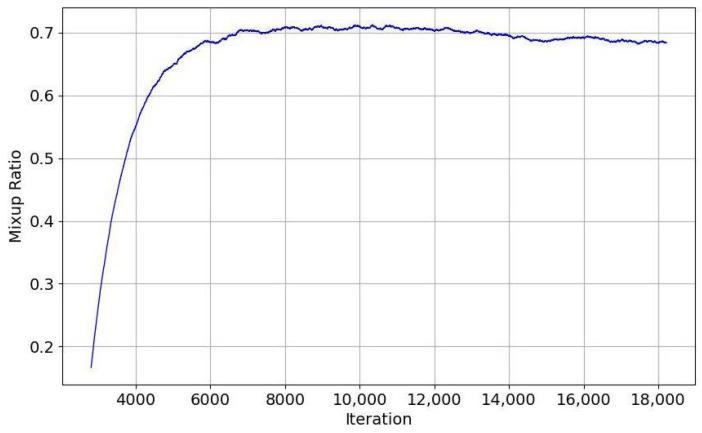
Mixup ratio curves for unlabeled target data.

Figure 3 demonstrates that the mixup ratio increases rapidly at first and then stabilizes at around 0.7 after 4000 iterations, indicating that the mixup ratio is adjusted during training to optimize model performance at a higher mixup ratio for the unlabeled target domain.

**Figure 4:**
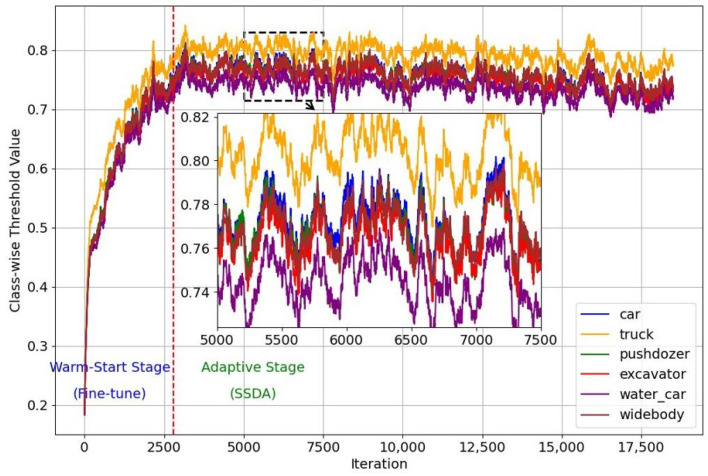
Class-wise pseudo-labeling threshold curves.

Figure 4 shows that the class-wise threshold varies with the number of iterations for different classes. In the warm-start phase, thresholds increase rapidly and stabilize after 2500 iterations. After entering the SSDA phase, thresholds fluctuate between 0.7 and 0.8, indicating that the adaptive thresholding strategy can quickly adjust the threshold of each category to a suitable range at the beginning of the SSDA phase.

After validating the contributions of each individual component through ablation studies, we next examined the effectiveness of our mixup strategies in more detail.

#### 3.5.1. Comparison with Random Mixup Ratio

Specifically, we compared our PMS against random mixup ratios sampled from a beta distribution with varying parameters (α values). Previous methods used a beta distribution Beta(α, α) to randomly sample mixup ratios between [0, 1], where the parameter α determined the shape of the distribution. For comparison, we selected three commonly used values of α (α=1, α=0.2, α=20).

As shown in Table 6, our PMS consistently outperformed the random mixup strategies. Specifically, the PMS achieved an mAP improvement of 1.5 over α=1, 1.4 over α=0.2, and 6.3 over α=20. These results demonstrate the PMS’s ability to better adapt to data requirements during training, leading to enhanced model generalization and performance on the object detection task.

#### 3.5.2. Comparison with Different Pseudo-Labeling Threshold Strategies

**Figure 5:**
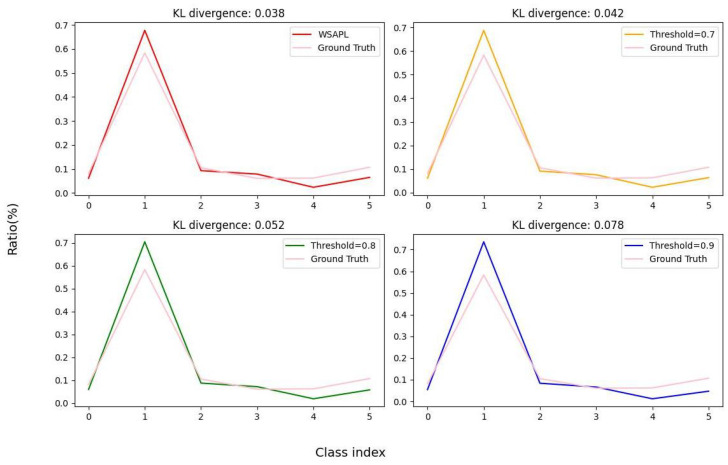
Class distributions of pseudo-labels generated by each model and KL divergence between the ground-truth label and pseudo-label distributions.

In addition to optimizing mixup strategies, we also compared our WSAPL strategy with three fixed-threshold methods (thresholds of 0.7, 0.8, and 0.9), and adaptive methods such as APL [29] and Freematch [30]. The results in Table 7 show that the WSAPL strategy achieved the highest mAP of 71.6, outperforming all of the methods compared. Of the fixed thresholds, 0.8 produced the best results, with an mAP of 71.1, while the other fixed thresholds resulted in an underperformance. WSAPL not only outperformed APL’s 70.5 but also significantly outperformed Freematch’s 68.9. Furthermore, in Figure 5, we compared the pseudo-label category distributions generated using the APL strategy and those from the three different fixed-threshold strategies against the true category distributions of the unlabeled target domain. By calculating the KL divergence among these distributions, we found that our APL strategy resulted in the smallest KL divergence, indicating that the pseudo-labels generated by our strategy most closely resemble the true distribution. These findings underscore the significant advantages of WSAPL in optimizing object detection performance, effectively adapting to varying data distributions, and addressing learning challenges across different categories.

### 3.6. Expansion and Data Efficiency Evaluation

In this section, we assess the expansion capability and data efficiency of Adamix when combined with active learning. We focus on its performance under different labeling budgets and its ability to reduce data requirements.

#### 3.6.1. Evaluation of Active Learning Expansion

To further validate the extensibility of our proposed Adamix method, we combined it with the active learning (AL) strategy and conducted comparative experiments with different active sampling budgets (5%, 7.5%, 15%, and 30%). The experimental results are shown in Table 8, where APT-AL represents the combination of the active learning approach in the APT study with its SSDA-OD approach, and Adamix-AL (our method) combines the active learning strategy in APT with our proposed Adamix approach. The experimental results indicate that Adamix-AL (our method) outperforms APT-AL across all labeling budgets. For example, with a 5% labeling budget, Adamix-AL achieves an maP of 76.7, which is significantly higher than APT-AL’s 73.8 mAP, and with labeling budgets of 7.5%, 15%, and 30%, Adamix-AL achieves maP values of 81.3, 87.4, and 88.4, outperforming APT-AL’s maP values of 78.3, 82.9, and 87.9, respectively. This indicates that our method demonstrates better performance across all labeling budgets.

Notably, with a 15% labeling budget, Adamix-AL surpassed the 86.4 mAP of the fully supervised Oracle method, and with a 30% labeling budget, Adamix-AL further improved to achieve an maP of 88.4. This demonstrates that when combined with active learning, our method can surpass the fully supervised Oracle method with an 85% reduction in labeling cost and outperform fully supervised models, reinforcing its ability to reduce the cost of annotation.

#### 3.6.2. Evaluation of Data-Saving Effectiveness

To further assess the data-saving potential of our method, experiments were conducted using 100%, 75%, 50%, and 40% of the target data, with active sampling budgets being set at 30% of the total number of training samples.

As shown in Table 9, when the target data were reduced to 75%, the model’s mAP decreased slightly to 87.0, which is still higher than that of the fully supervised Oracle (maP: 86.4). More importantly, when the target data were halved (50%), the model’s mAP was 86.8, indicating that it still outperformed Oracle. This shows that with the incorporation of active learning, our approach can reduce target data requirements by 50% and annotation requirements by up to 70%, significantly reducing the cost of annotation and target training data while maintaining high object detection performance. These results further validate our method’s effectiveness in reducing labeling costs and lowering real data requirements, especially in scenarios with high data acquisition and labeling costs.

### 3.7. Visualization

#### 3.7.1. Feature Visualization

**Figure 6:**
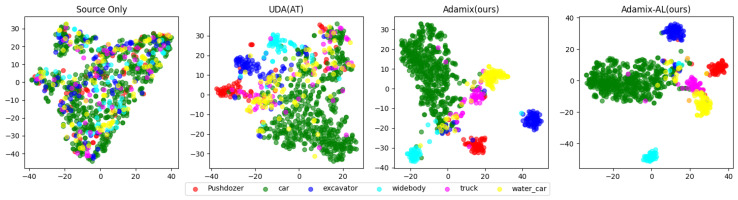
Visualization of instance features of different methods on Virtual-Mine to Actual-Mine via t-SNE.

We used t-SNE [39] to visualize the feature distributions of different methods in the target domain, as shown in Figure 6. In general, the Adamix-AL method (ours) performs optimally in terms of feature separation and its clustering effect, which is significantly better than that of the other methods.

The figure labeled “Source Only” shows a chaotic feature distribution, with no clear separation between different feature types, indicating the model’s difficulty in effectively differentiating various classes of features without any domain adaptation strategy. The UDA(AT) method improves feature distribution but still has a large number of feature overlaps. In contrast, the Adamix method (ours) achieves better intra-class aggregation and inter-class separation effects in the feature space, indicating better discriminability in dealing with different classes of features. Finally, Adamix-AL (our method) further enhances the clustering effect of features.

#### 3.7.2. Detection Result Visualization

**Figure 7:**
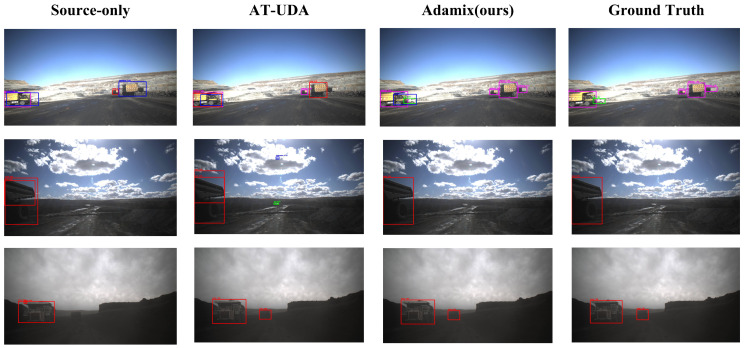

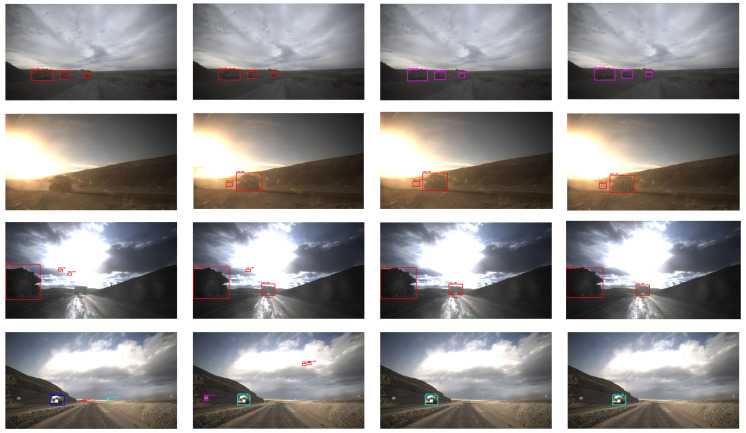
Detection results of different methods.

To compare the detection effectiveness of different algorithms under various weather conditions in an open-pit mining truck-driving scenario, we visualize the detection results of each method in Figure 7. From the results, we can see that our method’s results are the closest to the ground-truth labels.

Specifically, both the Source-Only and AT-UDA methods struggle with missing and falsely detecting objects. For instance, in the third and fifth rows of Figure 7, the Source-Only method misses distant trucks, especially in dusty and overexposed weather conditions. The AT-UDA method often misdetects the sky or ground shadows as other targets, although with fewer misses. In contrast, our proposed Adamix method significantly reduces both miss and false detection rates.

Furthermore, the Source-Only and AT-UDA methods often misidentify water cars and wide bodies, which are under-represented in the training data, as other objects, whereas the Adamix method accurately detects them, showing a substantial improvement in detecting long-tailed categories.

However, it is worth noting that even the Adamix method may exhibit detection errors for partially occluded or distant vehicles. For instance, in the first row of Figure 7, our method misses a car in the distance and incorrectly interprets a wide body on the left as a truck, indicating room for further improvement in complex scenarios.

## 4. Discussion

Despite its superior performance in the SSDA-OD task and the good scalability of combining ADA-OD with an active learning method, the proposed method, Adamix, still has several limitations. First, in our experimental setup, we uniformly defined real data of open-pit mines under different weather conditions as a single-target domain. However, the distribution of real data under different weather conditions varies greatly and should be considered different target domains. This transforms the original single-target domain adaptation problem into a multi-target domain adaptation [40] problem for object detection, a subject that has not been explored in depth in any related studies. To enhance the adaptability of the DA-OD method under diverse weather conditions, we plan to extend this work to a multi-target domain adaptation method for object detection. We will formulate corresponding domain adaptation and active learning strategies based on the characteristics of different target domains to fully exploit the potential of simulating data to solve the object detection problem in autonomous driving in open-pit mines under severe weather conditions.

Although valuable data were selected and virtual–real data were fully utilized in Adamix, research on data generation is relatively limited and currently relies solely on simulation engines. Recently, many data-driven data generation methods have emerged, such as the variational model, adversarial generation [41], diffusion modeling [42], and diffusion transformer [43] approaches. Based on these methods, we will further investigate data generation methods for data-driven autonomous driving. Dehazing methods [44,45,46] in unstructured open-pit mines will be combined with the SSDA-OD method proposed in this paper and an active learning method to construct a closed-loop training framework [47] for open-pit-mine driving data. By iteratively integrating data generation, valuable real-world sample mining, and automatic data labeling, an algorithm for autonomous driving in open-pit mines will be optimized.

## 5. Conclusions

This study presents a novel SSDA-OD method, Adamix, that adapts the mean teacher architecture to train an object detection model for open-pit mines. Progressive intermediate domain construction and warm-start adaptive pseudo-label are two key modules. The former generates intermediate domains during training to reduce source domain bias and overfitting for labeled target data; the latter calculates pseudo-label thresholds for initial classes by fine-tuning.

Experimental results obtained in a Sim2Real object detection scenario of an open-pit mine show that this method outperforms all baseline methods, significantly improving the domain adaptation performance of object detection. Specifically, Adamix achieves superior performance compared with fully supervised models, with only 50% of the annotation cost. Furthermore, integrating Adamix into an existing active domain adaptation strategy achieves fully supervised training performance with 50% real data and 30% of the annotation cost. This indicates that the proposed method, Adamix, weakly depends on real data and labeling costs in training object detection for autonomous driving trucks in open-pit mines.

Consequently, this method can speed up the deployment of autonomous truck systems in open-pit mines and improve their performance in unstructured environments. It also has the potential to be a key component in future closed-loop data systems for autonomous driving and can help overcome current simulation accuracy limitations.

## Figures and Tables

**Table 1:** Comparison of different methods’ mean average precision (mAP) in the Virtual-Mine to Actual-Mine domain adaptation scenario with different percentages (2.5%, 5%, 7.5%, 15%, 30%, and 50%) of labeled data.

	DA Methods	mAP					
		2.5%	5%	7.5%	15%	30%	50%
Source-Only	-	28.4	28.4	28.4	28.4	28.4	28.4
Supervised	-	23.4	40.0	45.6	59.9	68.0	80.0
AT-UDA [13]	UDA	40.2 ± 0.3	40.2 ± 0.3	40.2 ± 0.3	40.2 ± 0.3	40.2 ± 0.3	40.2 ± 0.3
CMT-UDA [10]		40.8 ± 0.4	40.8 ± 0.4	40.8 ± 0.4	40.8 ± 0.4	40.8 ± 0.4	40.8 ± 0.4
Co-training	SSDA	28.7 ± 0.4	40.2 ± 0.5	47.3 ± 0.8	63.0 ± 0.6	73.6 ± 0.5	81.1 ± 1.2
AT-SSDA [13]		40.7 ± 0.6	44.6 ± 0.3	54.5 ± 0.7	68.3 ± 0.5	78.6 ± 0.9	84.5 ± 0.7
CMT-SSDA [10]		42.7 ± 0.3	45.6 ± 0.5	55.5 ± 0.4	68.8 ± 0.6	80.1 ± 0.8	84.7 ± 0.5
Dual-Teacher [31]		52.1 ± 0.1	57.8 ± 0.4	63.5 ± 0.3	71.7 ± 0.5	80.6 ± 0.6	84.9 ± 0.8
APT-SSDA [9]		53.6 ± 0.2	62.0 ± 0.3	68.2 ± 0.3	73.9 ± 0.6	83.2 ± 0.8	85.1 ± 0.6
Adamix (our method)		57.0± 0.1	63.6 ± 0.6	71.6 ± 0.2	76.8 ± 0.3	83.7 ± 0.4	86.4 ± 0.3
Oracle	-	86.4	86.4	86.4	86.4	86.4	86.4

**Table 2:** Ablation studies on each component of our method.

PIDC	WSAPI	mAP
		68.2 ± 0.5
	✓	70.2 ± 0.2
✓		69.9 ± 0.3
✓	✓	71.6 ± 0.2

**Table 3:** Ablation studies for $\lambda_{1}$.

$\lambda_{1}=0.9$	$\lambda_{1}=0.99$	$\lambda_{1}=0.999$	$\lambda_{1}=0.9999$
68.3	70.3	71.6	69.6

**Table 4:** Ablation studies for $\lambda_{2}$.

$\lambda_{2}=0.9$	$\lambda_{2}=0.99$	$\lambda_{2}=0.999$	$\lambda_{2}=0.9999$
68.9	71.6	70.4	67.2

**Table 5:** Ablation studies for η.

Category-Specific Confidence	η=0.1	η=0.2	η=0.3
67.2	71.6	70.7	70.1

**Table 6:** A comparison of our PMS with different mixup strategies. The α values represent the parameter of the beta distribution used for randomly sampling the mixup ratio.

α = 1	α = 0.2	α = 20	PMS (Our Strategy)
70.1	70.2	65.3	71.6

**Table 7:** Comparison of our WSAPL with other threshold strategies, where 0.7, 0.8, and 0.9 represent methods using fixed thresholds, which are commonly employed by mean teacher models [10,13,38].

0.7	0.8	0.9	APL [23]	Freematch [24]	WSAPL (Ours)
69.4	71.1	70.2	70.5	68.9	71.6

**Table 8:** Comparison of our method, Adamix-AL, with APT-AL under different labeling budgets (5%, 7.5%, 15%, 30%), when expanded with active learning.

	5%	7.5%	15%	30%
APT-AL [5]	73.8	78.3	82.9	87.9
Adamix-AL (our method)	76.7	81.3	87.4	88.4

**Table 9:** The performance of Adamix-AL under different data utilization rates, with 30% of the real data being actively selected for labeling.

100%	75%	50%	40%
88.4	87	86.8	84.7

## Data Availability

The Virtual-Mine and Actual-Mine datasets were constructed in our previous work. These datasets are private and can be made available upon request from the corresponding author.

## References

[1] Cerna G.E.P., Hernández J.R.C., Herazo J.C.M., Castillo A.P. (2023). Evaluation of the overall effectiveness (OEE) of autonomous transportation system (AHS) equipment and its impact on mine design. Open pit mine case study. Procedia Comput. Sci..

[2] Chen L., Li Y., Li L., Qi S., Zhou J., Tang Y., Yang J., Xin J. (2024). High-Precision Positioning, Perception and Safe Navigation for Automated Heavy-Duty Mining Trucks. IEEE Trans. Intell. Veh..

[3] Voronov Y., Voronov A., Makhambayev D. (2020). Current State and Development Prospects of Autonomous Haulage at Surface Mines. Proceedings of the E3S Web of Conferences.

[4] Guo L., Zhang Y., Liu J., Liu H., Li Y. (2025). Scale-Consistent and Temporally Ensembled Unsupervised Domain Adaptation for Object Detection. Sensors.

[5] Guo Y., Yu G., Zhou B., Wang Y., Liu G. Research on Open-pit Mine Virtual Environment Construction and Working Vehicle Modeling Simulation Based on PreScan. Proceedings of the 2020 Chinese Intelligent Systems Conference.

[6] Ai Y., Liu Y., Gao Y., Zhao C., Cheng X., Han J., Tian B., Chen L., Wang F.-Y. (2024). PMWorld: A Parallel Testing Platform for Autonomous Driving in Mines. IEEE Trans. Intell. Veh..

[7] Johnson-Roberson M., Barto C., Mehta R., Sridhar S.N., Rosaen K., Vasudevan R. Driving in the Matrix: Can Virtual Worlds Replace Human-Generated Annotations for Real World Tasks?. Proceedings of the IEEE International Conference on Robotics and Automation (ICRA), Marina Bay Sands.

[8] Ros G., Sellart L., Materzynska J., Vazquez D., Lopez A.M. The SYNTHIA Dataset: A Large Collection of Synthetic Images for Semantic Segmentation of Urban Scenes. Proceedings of the IEEE Conference on Computer Vision and Pattern Recognition (CVPR).

[9] Guo L., Guo Y., Ai Y., Ge S. (2024). Active Parallel Teacher for Human-in-the-Loop Sim2Real Object Detection in Autonomous Haulage Trucks. IEEE Trans. Intell. Veh..

[10] Cao S., Joshi D., Gui L.-Y., Wang Y.-X. Contrastive Mean Teacher for Domain Adaptive Object Detectors. Proceedings of the IEEE/CVF Conference on Computer Vision and Pattern Recognition (CVPR).

[11] Yu F., Wang D., Chen Y., Karianakis N., Shen T., Yu P., Lymberopoulos D., Lu S., Shi W., Chen X. SC-UDA: Style and Content Gaps Aware Unsupervised Domain Adaptation for Object Detection. Proceedings of the IEEE/CVF Winter Conference on Applications of Computer Vision (WACV).

[12] Chen Y., Li W., Sakaridis C., Dai D., Van Gool L. Domain Adaptive Faster R-CNN for Object Detection in the Wild. Proceedings of the IEEE Conference on Computer Vision and Pattern Recognition (CVPR).

[13] Li Y.-J., Dai X., Ma C.-Y., Liu Y.-C., Chen K., Wu B., He Z., Kitani K., Vajda P. Cross-domain Adaptive Teacher for Object Detection. Proceedings of the IEEE/CVF Conference on Computer Vision and Pattern Recognition (CVPR).

[14] Weng W., Yuan C. Mean Teacher DETR with Masked Feature Alignment: A Robust Domain Adaptive Detection Transformer Framework. Proceedings of the AAAI Conference on Artificial Intelligence.

[15] Nakamura Y., Ishii Y., Yamashita T. Active Domain Adaptation with False Negative Prediction for Object Detection. Proceedings of the IEEE/CVF Conference on Computer Vision and Pattern Recognition (CVPR).

[16] Menke M., Wenzel T., Schwung A. (2024). Bridging the Gap: Active Learning for Efficient Domain Adaptation in Object Detection. Expert Syst. Appl..

[17] Saito K., Kim D., Sclaroff S., Darrell T., Saenko K. Semi-Supervised Domain Adaptation via Minimax Entropy. Proceedings of the IEEE/CVF International Conference on Computer Vision.

[18] Xiao J., Dai Q., Shen X., Xie X., Dai J., Lam J., Kwok K.W. (2024). Semi-Supervised Domain Adaptation on Graphs with Contrastive Learning and Minimax Entropy. Neurocomputing.

[19] Chen S., Jia X., He J., Shi Y., Liu J. Semi-Supervised Domain Adaptation Based on Dual-Level Domain Mixing for Semantic Segmentation. Proceedings of the International Conference on Computer Vision and Pattern Recognition (CVPR).

[20] Kim D., Seo M., Park K., Shin I., Woo S., Kweon I.S., Choi D.G. Bidirectional Domain Mixup for Domain Adaptive Semantic Segmentation. Proceedings of the AAAI Conference on Artificial Intelligence.

[21] Li K., Wang S., Yu L., Heng P.A. (2020). Dual-Teacher++: Exploiting Intra-Domain and Inter-Domain Knowledge with Reliable Transfer for Cardiac Segmentation. IEEE Trans. Med. Imaging.

[22] Liu X., Xing F., Shusharina N., Lim R., Jay Kuo C.C., El Fakhri G., Woo J. ACT: Semi-Supervised Domain-Adaptive Medical Image Segmentation with Asymmetric Co-Training. Proceedings of the International Conference on Medical Image Computing and Computer-Assisted Intervention.

[23] Luo C., Zhang Y., Guo J., Hu Y., Zhou G., You H., Ning X. (2024). SAR-CDSS: A Semi-Supervised Cross-Domain Object Detection from Optical to SAR Domain. Remote Sens..

[24] Arruda V.F., Berriel R.F., Paixão T.M., Badue C., De Souza A.F., Sebe N., Oliveira-Santos T. (2022). Cross-Domain Object Detection Using Unsupervised Image Translation. Expert Syst. Appl..

[25] Zhang G., Wang L., Chen Z. (2024). A Step-Wise Domain Adaptation Detection Transformer for Object Detection under Poor Visibility Conditions. Remote Sens..

[26] Zhuang C., Han X., Huang W., Scott M. iFAN: Image-Instance Full Alignment Networks for Adaptive Object Detection. Proceedings of the AAAI Conference on Artificial Intelligence.

[27] Jin M., Li K., Li S., He C., Li X. Towards Realizing the Value of Labeled Target Samples: A Two-Stage Approach for Semi-Supervised Domain Adaptation. Proceedings of the IEEE International Conference on Acoustics, Speech and Signal Processing (ICASSP).

[28] Chang H., Chen K., Wu M. A Two-stage Cascading Method Based on Finetuning in Semi-supervised Domain Adaptation Semantic Segmentation. Proceedings of the Asia-Pacific Signal and Information Processing Association Annual Summit and Conference (APSIPA ASC).

[29] Nie Y., Fang C., Cheng L., Lin L., Li G. Adapting Object Size Variance and Class Imbalance for Semi-supervised Object Detection. Proceedings of the AAAI Conference on Artificial Intelligence.

[30] Wang Y., Chen H., Heng Q., Hou W., Fan Y., Wu Z., Wang J., Savvides M., Shinozaki T., Raj B. FreeMatch: Self-adaptive Thresholding for Semi-supervised Learning. Proceedings of the International Conference on Learning Representations.

[31] Zheng X., Cui H., Xu C., Lu X. (2023). Dual Teacher: A Semisupervised Cotraining Framework for Cross-Domain Ship Detection. IEEE Trans. Geosci. Remote Sens..

[32] Yang L., Wang Y., Gao M., Shrivastava A., Weinberger K.Q., Chao W.L., Lim S.N. Deep Co-Training With Task Decomposition for Semi-Supervised Domain Adaptation. Proceedings of the IEEE/CVF International Conference on Computer Vision (ICCV).

[33] Zhang H., Cisse M., Dauphin Y.N., Lopez-Paz D. mixup: Beyond Empirical Risk Minimization. Proceedings of the International Conference on Learning Representations.

[34] Li J., Li G., Yu Y. (2024). Inter-domain mixup for semi-supervised domain adaptation. Pattern Recognit..

[35] Jing M., Meng L., Li J., Zhu L., Shen H.T. (2023). Adversarial Mixup Ratio Confusion for Unsupervised Domain Adaptation. IEEE Trans. Multimed..

[36] Ren S., He K., Girshick R., Sun J. (2016). Faster R-CNN: Towards Real-Time Object Detection with Region Proposal Networks. IEEE Trans. Pattern Anal. Mach. Intell..

[37] Li Y., Li Z., Teng S., Zhang Y., Zhou Y., Zhu Y., Cao D., Tian B., Ai Y., Xuanyuan Z. AutoMine: An Unmanned Mine Dataset. Proceedings of the IEEE/CVF Conference on Computer Vision and Pattern Recognition (CVPR).

[38] Liu Y.C., Ma C.Y., He Z., Kuo C.W., Chen K., Zhang P., Wu B., Kira Z., Vajda P. Unbiased Teacher for Semi-Supervised Object Detection. Proceedings of the International Conference on Learning Representations (ICLR).

[39] Van der Maaten L., Hinton G. (2008). Visualizing data using t-SNE. J. Mach. Learn. Res..

[40] Isobe T., Jia X., Chen S., He J., Shi Y., Liu J., Lu H., Wang S. Multi-Target Domain Adaptation with Collaborative Consistency Learning. Proceedings of the IEEE/CVF Conference on Computer Vision and Pattern Recognition.

[41] Shivashankar C., Miller S. Semantic Data Augmentation With Generative Models. Proceedings of the IEEE/CVF Conference on Computer Vision and Pattern Recognition (CVPR).

[42] Wang Y., He J., Fan L., Li H., Chen Y., Zhang Z. Driving into the Future: Multiview Visual Forecasting and Planning with World Model for Autonomous Driving. Proceedings of the IEEE/CVF Conference on Computer Vision and Pattern Recognition (CVPR).

[43] Peebles W., Xie S. Scalable Diffusion Models with Transformers. Proceedings of the IEEE/CVF International Conference on Computer Vision (ICCV).

[44] Liu Y., Yan Z., Ye T., Wu A., Li Y. (2022). Single Nighttime Image Dehazing Based on Unified Variational Decomposition Model and Multi-Scale Contrast Enhancement. Eng. Appl. Artif. Intell..

[45] Zhang S., Ren W., Tan X., Wang Z.-J., Liu Y., Zhang J., Zhang X., Cao X. (2021). Semantic-Aware Dehazing Network with Adaptive Feature Fusion. IEEE Trans. Cybern..

[46] Zhang S., Zhang X., Wan S., Ren W., Zhao L., Shen L. (2023). Generative Adversarial and Self-Supervised Dehazing Network. IEEE Trans. Ind. Inform..

[47] Li X., Wang Z., Huang Y., Chen H. (2023). A Survey on Self-Evolving Autonomous Driving: A Perspective on Data Closed-Loop Technology. IEEE Trans. Intell. Veh..

